# European Society of Sexual Medicine consensus statement on the use of animal models for studying Peyronie’s disease

**DOI:** 10.1093/sexmed/qfad046

**Published:** 2023-08-02

**Authors:** Fabio Castiglione, Onur Ö Çakır, Nicolò Schifano, Giovanni Corona, Yacov Reisman, Carlo Bettocchi, Selim Cellek, Marcus M Ilg

**Affiliations:** King’s College London Hospital, London SE5 9RS, United Kingdom; King’s College London Hospital, London SE5 9RS, United Kingdom; King’s College London Hospital, London SE5 9RS, United Kingdom; Endocrinology Unit, Medical Department, Maggiore-Bellaria Hospital, Azienda USL, Bologna 40139, Italy; Flare-Health, Amstelveen 1012, the Netherlands; Department of Urology, University of Bari, Bari 70121, Italy; Fibrosis Research Group, Medical Technology Research Centre, Anglia Ruskin University, Chelmsford, Essex CM1 1SQ, United Kingdom; Fibrosis Research Group, Medical Technology Research Centre, Anglia Ruskin University, Chelmsford, Essex CM1 1SQ, United Kingdom

**Keywords:** Peyronie’s disease, animal model, rat, mouse, rabbit, fibrosis

## Abstract

**Introduction:**

Animal models are frequently used for translational research in Peyronie’s disease (PD). However, due to lack of availability of guidelines, there is some heterogeneity in study design, data reporting, and outcome measures.

**Aim:**

This European Society for Sexual Medicine consensus statement aims to provide guidance in utilization of animal models in PD research in a standardized and uniform fashion.

**Methods:**

PubMed was searched for studies using animal models for PD. The following search terms were used: (“Peyronie’s disease” OR “penile fibrosis” OR “penile curvature” OR “induration penis plastica” OR “erectile dysfunction”) AND (“rodent” OR “mouse” OR “mice” OR “rat” OR “rabbit”).

**Outcomes:**

This European Society for Sexual Medicine statement describes best practice guidelines for utilization of animals in PD research: power calculation, details of available models, surgical procedures, and measurement techniques, while highlighting possible pitfalls and translational limitations of the models.

**Results:**

In total, 2490 studies were retrieved and 2446 articles were excluded. A total of 44 studies were included, of which 40 studies used rats, 1 study used both rats and mice, 1 study used a genetic mouse model, and 2 studies used rabbits. A significant number of the studies (70.5%) used transforming growth factor β 1 for induction of fibrosis. Oxford 2011 Levels of Evidence criteria could not be applied due to the nature of the studies.

**Conclusion:**

Despite certain limitations of PD animal models presented, we aimed to provide guidance for their appropriate use in translational research, with the purpose of improving study quality and reproducibility as well as facilitating interpretation of reported results and conclusions.

## Introduction

Peyronie’s disease (PD) is characterized by localized fibrotic plaque formation in the penile tunica albuginea (TA)^1^ with a prevalence of 0.3% to 9%.^2^^,^^3^ PD presents in 2 phases. First, an acute (unstable) phase, which can present with pain during erection and is characterized by progressive development of the fibrotic plaque. This is followed by a chronic phase that begins to stabilize 12 to 18 months after the first symptoms, along with improvement of pain with a stable penile plaque and curvature.^4^ Permanent curvature or plaque induration can have drastic consequences, such as difficulties to penetrate, in some cases severe erectile dysfunction, and can affect the quality of life.^4–6^

Although the etiology of PD is not clear, repetitive microtrauma to the TA, caused by not fully rigid erection, is generally accepted to be the trigger for inflammation which leads to aberrant wound healing and extracellular matrix (ECM) protein (mostly collagen and elastin) deposition and disorganization. In the chronic phase of PD, this can even lead to calcification of the plaque.^7^^,^^8^ In addition, a genetic predisposition with other fibrotic disorders such as Dupuytren’s disease has been suggested, along with a single nucleotide polymorphism (G915C) in the gene for transforming growth factor beta 1 (TGF-β1).^9^^,^^10^ Regardless of the triggers, a common feature of all fibrotic diseases, including PD, is the activation of ECM-producing myofibroblasts, which are the key mediators of fibrotic tissue remodeling.^11^^,^^12^

In an effort to understand the pathophysiology of PD and to develop novel medicines to treat the condition, animal models that resemble the human condition have been developed. Mouse, rat, and rabbit models have been utilized to study PD in vivo.^11^^,^^13–55^ Most models rely on inducing fibrosis in TA either by increasing TGF-β1 in TA or by surgical trauma of the TA. The Tight-skin 1 (Tsk) model is the only genetic model for the disease in which PD-like fibrosis develops spontaneously.^38^

Multiple preclinical studies have used PD animal models to investigate the antifibrotic efficacy of various molecules and treatments.^56^ Endpoints typically include improved cavernosal smooth muscle/collagen ratios on immunohistochemical staining or messenger RNA and protein content of fibrosis-related genes and proteins such as α-smooth muscle actin, collagens, and elastin.^57^ Moreover, erectile function assessments are often included by measuring intracavernous pressure (ICP) in response to cavernous nerve electrostimulation.^48^

However, despite the promising results from preclinical studies, most clinical trials have failed to confirm any structural or lasting benefit of the treatments that were efficacious in animal models in improving the curvature in PD patients, meaning that most of these animal studies failed to translate to the clinical outcomes.^58–60^ Recently, a preclinical study that utilized TGF-β1–induced PD model in rats^39^ showed promising initial results in the clinic.^61^^,^^62^ Poor design of both clinical and preclinical studies could be partly responsible for this lack of translation. Another cause could be the high variance in methodologies and subsequent reported outcomes when conducting animal studies. A similar discrepancy has been noted in other animal models used in the sexual medicine field, such as the cavernous nerve injury model. This model uses similar techniques and outcome measurements as in PD.^63^ These methodological inconsistencies and the lack of consensus guidelines surrounding this topic raise the concern of an increasing number of studies being published with nonreproducible results, leading to little progress in the field.

The aim of this statement paper is to review the current state of art, highlight possible pitfalls, and provide guiding statements for experimental design, technique, and reporting of results when using animal models of PD and to provide further standardization and improvement in the quality of research in this field on behalf of the European Society of Sexual Medicine (ESSM).

## Methods

### Literature search and study eligibility

A literature search of full-text English language publications on basic science studies using an in vivo rodent animal model for PD was performed using PubMed on July 30, 2020, and repeated on February 23, 2023, with the search term (“Peyronie’s disease” OR “penile fibrosis” OR “penile curvature” OR “induration penis plastica” OR “erectile dysfunction”) AND (rodent OR mouse OR mice OR rat OR rabbit).

Inclusion criteria were

peer-reviewed publications with primary data,

English language,

studies using an in vivo animal model of PD, and

studies with the aim to develop or evaluate an animal model for PD or to test a treatment using an animal model for PD.

Exclusion criteria were

a secondary data research such as abstracts, letters, and reviews,

studies using an in vivo animal model of fibrosis of penile corpus cavernosum only, and

studies using an in vivo animal model in which TA grafts are investigated.

### Data extraction

The studies were reviewed by 2 different persons (O.O.C., F.C.). If it was not clear from the abstract whether the article might contain relevant data, the full article was assessed.

### Review methods

Thereafter, relevant studies were identified, analyzed, and summarized after an interactive peer-review process by the entire panel to obtain a narrative review. The statements were discussed among the panel members. Disagreements were resolved by consensus. It was not possible to grade the studies according to the Oxford 2011 Levels of Evidence criteria^64^ because of the nature (basic science) of the studies. The ESSM position is summarized in the specific statements.

## General observations

Of 2490 studies retrieved, 2446 articles were excluded. A total of 44 studies were included in this review^11^^,^^13–53^ which are summarized in Table 1. Among them, 40 studies used rats only, 1 study used both rats and mice, 1 study used a genetic mouse model, and 2 studies used rabbits (Table 1). A total of 31 (70.5%) of the 44 studies used either injection of TGF-β1 into TA or increased TGF-β1 levels using viruses or plasmids. The remaining studies used one of the following methods: surgical trauma to the TA, TA injection of fibrin, thrombin, chlorhexidine, allograft of TA tissue, extracorporeal shockwave, human PD cells, or rat blood or plasma (Table 1).

**Table 1:** Summary of 44 studies identified.

Study	Species; number of animals per group; age; weight	Negative control group	Inducer and dosage	Type of treatment, route and dosage	Time of treatment start	Erectile function evaluation	Time of evaluation	Methods of evaluation
El Sakka et al, 1997^52^	SD rats; 6 rats per group; age and weight not documented	YES (injection of saline into TA)	Injection of cytomodulin (a synthetic peptide with TGF-β1–like activity; 10, 100, and 100 nmol/L) into TA, once	None	N/A	NO	3 d, 2 wk, and 6 wk	MTs, Hart’s stain, PCR (TGF-β1)
El Sakka et al, 1998a^51^	SD rats; 6 rats per group; age and weight not documented	YES (sham surgery)	Surgical trauma to TA, once	None	N/A	NO	6 hours, 1 and 3 d, and 8 wk	MTs, Hart’s stain, EM, WB (TGF-β1)
El Sakka et al, 1998b^50^	SD rats; 6 rats per group; age and weight not documented	YES (injection of saline into TA)	Injection of cytomodulin (10, 100, and 100 nmol/L) into TA, once	None	N/A	NO	3 d, 2 wk, and 6 wk	MTs, Hart’s stain, EM
El Sakka et al, 1999^49^	SD rats; 6 rats per group; age and weight not documented	NO	Injection of TGF-β1 (1000 nM/0.1 mL) into TA, once	Colchicine (0.035 mg/kg/d oral or ibuprofen 17 mg/kg/d oral), once daily	Day 0 and week 6	NO	6 and 12 wk	MTs, Hart’s stain, EM, WB (TGF-β1)
Bivalacqua et al, 2000^48^	CD rats; 6 rats per group; weight (275-350 g); age not documented	YES (injection of saline into TA)	Injection of TGF-β1 (0.5 μg/100 μL) into TA or surgical trauma to TA, once	Aminoguanidine (iNOS inhibitor) 5 mg/kg i.v. during ICP measurements	Day 0	YES (ICP-cavNS)	Day 2, 3 wk, and 6 wk	MTs, Hart’s stain
Bivalacqua et al, 2001^47^	CD rats; 8-10 rats per group; weight 300 g; age 10 wk	YES (injection of saline into TA)	Injection of TGF-β1 (0.5 μg/100 μL) into TA, once	None	N/A	NO	6 wk	MTs, Hart’s stain
Ferrini et al. 2002^46^	Fisher 344 rats; 5 rats per group; weight not documented; age 9-11 mo	YES (injection of saline into TA)	Injection of TGF-β1 (0.5 μg) into TA, once	L-NIL (nitric oxide synthase inhibitor) 100 mg/L in drinking water for 45 d	Day 0	NO	Day 45	MTs, Hart’s stain, Sirius red
Vernet et al, 2002^45^	Fisher 344 rats; 5 rats per group; weight not documented; age 9-11 mo	YES (injection of saline into TA)	Injection of TGF-β1 (0.5 μg) into TA, once	L-NIL (nitric oxide synthase inhibitor) 100 mg/L in drinking water for 45 d	Day 0	NO	Day 45	IHC (vimentin and α-SMA), Collagen I (using gene promotor)
Davila et al, 2003^44^	SD rats; 3-5 rats per group; weight not documented; age 9-11 mo	YES (injection of saline or rat IgGs or human serum proteins into TA)	Injection of fibrin (30 μL) with or without TGF-β1 (0.5 μg/60 μL) into TA, once	None	N/A	NO	3 and 6 wk	MTs, VVG stain, Hart’s stain, IHC (α-SMA, TGF-β1), EM
Valente et al, 2003^43^	Fisher 344 rats; 5 rats per group; weight not documented; age 9-11 mo	YES (injection of saline into TA)	Injection of TGF-β1 (0.5 μg) into TA, once	Pentoxifylline (10 mg/kg/d) or sildenafil (10 mg/kg/d) or L-arginine (2.25 g/kg/d) in drinking water for 45 d	Day 0	NO	Day 45	MTs, IHC (vimentin, collagen and α-SMA)
Davila et al, 2004^42^	Balb mice and SD rats; 5 animals per group; weight not documented; mice 3 mo old, rats 8-11 mo old	NO	Injection of fibrin (30-60 μL) into TA, once	Plasmid-expressing iNOS, injection into TA	Days 21 and 32	NO	Day 45	MTs, IHC (TGF-β1)
Ferrini et al 2006^41^	Fisher 344 rats; 8 rats per group; weight not documented; age 9-11 mo	YES (injection of saline into TA)	Injection of TGF-β1 (0.5 μg) into TA, once	Vardenafil (0.4-1.2 mg once daily; 10-30 mg/L in drinking water) for 45 or 87 d	Days 0 and 45	NO	Days 45 and 87	MTs, Picrosirius red staining, IHC (α-SMA and TGF-β1)
Piao et al, 2007^40^	SD rat; 18 rats per group; weight not documented; age 4 mo	YES (saline or LacZ adenovirus injection into TA)	Injection of TGF-β1 (0.7 μg/100 μL) or adenovirus (1-3 × 10^10^ particles/100 μL) encoding porcine TGF-β1 into TA; single dose or repeated 3 times on days 0, 3, and 6	None	N/A	YES (ICP-cavNS)	Days 30, 45, and 60	MTs, Hart’s stain, H&E, Safranin-O stain, IHC (TGF-β1), Penile curvature
Cantini et al, 2008^53^	Fisher 344 rats; 5 rats per group; weight not documented; age 8-11 mo	YES (injection of saline into TA)	Injection of TGF-β1 (0.5 μg) into TA, once	Adenoviruses (2 × 10^6^ ivu) encoding myostatin or myostatin short hairpin RNA on day 0 or suppressing myostatin on week 5	Day 0 or week 5	NO	Day 45	MTs
Lucattelli et al, 2008^38^	B6.CgFbn1Tsk+/+Pldnpa/J mice (tight skin mice); 8 mice per group; weight not documented; age 2-24 mo	YES (C57Bl/6 J wild type mice)	A mouse model that develops fibrosis spontaneously	None	N/A	NO	2, 4, 6, 8, 10, 12, and 24 mo of age	MTs, H&E, HP assay, IHC (TGF-β1, collagens), PCR (TGF-β1, collagens)
Andrade et al. 2009^37^	NZW rabbits; 5 rabbits per group; age and weight not documented	YES (no shockwave group and a sham group)	Extracorporeal shockwave; applied in 3 sessions	None	N/A	NO	Days 0 and 7	H&E
Ryu et al, 2009^36^	SD rats; 10 rats per group; weight not documented; age 4 mo	YES (no treatment)	Injection of adenovirus (1 × 10^10^ particles/0.1 mL) expressing TGF-β1 into TA on days 0, 3, and 6	IN-1130 (TGF-β1 receptor kinase [activin receptor-like kinase 5] inhibitor) 5 mg/kg; injection into TA on days 30 and 37	Days 30 and 37	YES (ICP-cavNS)	Day 45	MTs, Hart’s stain, Safranin-O stain, IHC (TGF-β1), PCR (TGF-β1), HP assay
Akman et al, 2013^35^	SD rats; 8-9 rats per group; weight 300-400 g; age 12 wk	YES (injection of bovine serum albumin into TA)	Injection of TGF-β1 (0.5 μg/0.25 mL) into TA, once	Decorin (a proteoglycan involved in collagen synthesis and sequesters TGF-β1) 0.5 μg/0.25 mL, injection into CC, 4 times for 4 consecutive days	Day 2	YES (ICP-cavNS)	6 wk	H&E
Castiglione et al, 2013^34^	SD rats; 9 rats per group; weight 300-350 g; age 12 wk	YES (injection of citrate buffer into TA)	Injection of TGF-β1 (0.5 μg/50 μL) into TA, once	Human adipose tissue–derived stem cells, 1 × 10^6^ cells, injection into TA	Day 1	YES (ICP-cavNS)	5 wk	MTs, WB (collagens, elastin, tubulin), IHC (collagens, α-SMA, elastin)
Cerruto et al, 2013^33^	Wistar rats; 4 rats in total; weight 420-450 g; age not documented	NO	Injection of thrombin (amount undocumented) into TA, once	None	N/A	NO	Days 7 and 21	magnetic resonance imaging
Chung et al. 2013^32^	SD rats; 2-5 rats per group; weight not documented; age 9-11 mo	YES (no treatment)	Injection of TGF-β1 (0.1 mL) + sodium tetradecyl sulfate (a sclerosing agent, 0.1 mL 3%) into TA, once	Verapamil (0.1-0.5 mg/0.1 mL every second day for 2 wk), injection into TA	4 wk	YES (ICP-cavNS)	6 and 8 wk	MTs Hart’s stain IHC (TGF-β1, α-SMA, collagenase)
Ferretti et al 2014^55^	SD rats; 8 rats per group; weight not documented; age 16 wk	YES (autograft of TA)	Allograft over surgically-induced TA defect	None	N/A	YES (ICP-cavNS)	1, 3, 7, and 12 wk	MTs, Von Kossa stain, Hart’s stain, IHC (Smad2, collagen, osteocalcin), penile curvature
Gelfand et al, 2014^11^	Fisher 344 rats; 5 rats per group; weight not documented; age 8-11 mo	YES (injection of saline into TA)	Injection of human fibroblasts (1 × 10^6^ cells) obtained from PD patients into TA, once	None	N/A	NO	2 and 4 wk	MTs, H&E, Picrosirius staining, IHC (collagen), WB (α-SMA, calponin), HP assay, PCR
Gokce et al, 2014^31^	SD rats; 6 rats per group; weight 300-350 g; age not documented	YES (injection of saline into TA)	Injection of TGF-β1 (0.5 μg) into TA, once	Rat adipose-derived stems cells; 5 × 10^5^ cells, injection into TA	Days 0 and 30	YES (ICP-cavNS)	Day 45	MTs, H&E, ZyA (MMPs), PCR (TIMPs, MMPs)
Kwon et al 2014^54^	SD rats; 6 rats per group; weight not documented; age 4 mo	YES (no treatment and control adenovirus)	Injection of human fibrin and thrombin solutions (100 μL each) on days 0 and 5	Injection of adenovirus encoding histone deacetylase small hairpin RNA (1 × 10^8^ pfu/0.1 mL) into TA on day 15	Day 15	NO	Day 30	MTs, H&E, IHC (vimentin, p-Smad3)
Sohn et al. 2014^30^	SD rats; 8 rats per group; weight 250-350 g; age not documented	YES (injection of saline into TA)	Injection of fibrin and thrombin mixture (30 μL) into TA, once	Anthocyanin (a natural pigment; 50 mg/kg orally twice a day for 4 wk)	Day 0	NO	Day 30	MTs, IHC (TGF-β1)
Gokce et al, 2015^29^	SD rats; 6 rats per group; weight 300-350 g; age 12 wk old	YES (injection of saline into TA)	Injection of TGF-β1 (0.5 μg/50 μL) into TA, once	Rat adipocyte-derived stems cells (5 × 10^5^ cells) expressing IFNα-2b; injection into TA	Days 0 and 30	YES (ICP-cavNS)	Day 45	MTs, H&E, VVG stain, ZyA (MMPs), PCR (TIMPs and MMPs)
Kaya et al, 2017^28^	SD rats; 4-7 rats per group; weight 350-470 g; age 12 wk	YES (injection of bovine serum albumin into TA)	Injection of TGF-β1 (0.5 μg in 250 μL) into TA, once	Intracavernosal mitomycin-C (0.3 mg/mL) once daily on days 2, 3, 4, and 5	Day 2	NO	6 wk	MTs, H&E, VVG
Lin et al 2017^27^	SD rats; 10 rats per group; age and weight not documented	NO	Injection of TGF-β1 (0.1 mL) + sodium tetradecyl sulfate (a sclerosing agent, 0.1 mL 3%) into TA, once	Vacuum erection device (5 cycles/d) and traction device (3 cycles/d) from week 4 for 4 wk	Week 4	YES (ICP-cavNS)	8 wk	H&E, IHC (TGF-β1, α-SMA, Smad2/3)
Cohen et al, 2018^26^	Wistar rats; 5 rats per group; weight 340 g; age 12 wk	YES (injection of distilled water into TA)	Injection of autologous blood (20 μL) into TA, once	None	N/A	NO	Days 14 and 45	MTs, H&E, Picrosirius stain, IHC (TGF-β1, MMP, collagen),
Li et al. 2018^25^	SD rats; 9 rats per group; weight 280-320 g; age not documented	YES (saline injection into TA)	Injection of TGF-β1 (0.5 μg/50 μL) into TA, once	Vacuum erection device from day 32 for 10 d	Day 32	YES (ICP-cavNS)	Day 42	MTs, IHC (TGF-β1, Smad2), EM, WB (TGF-β1, Smad2)
Culha et al, 2018^24^	SD rats; 5 rats per group; weight 300-350 g; 9-11 mo	YES (saline injection into TA)	Injection of TGF-β1 (0.1 mL) into TA, once	Injection of autologous platelet-rich plasma (0.1 mL) into TA	Day 0 and 15	NO	Day 45	H&E, MTs, Picrosirius stain
Jiang et al, 2018^23^	SD rats, 5-6 rats per group; weight and age not documented	YES (saline injection into TA)	Injection of 0.1% chlorhexidine gluconate plus 15% ethanol dissolved in saline into TA; 5 repeats on days 0, 7, 14, 21, and 28	None	N/A	YES (ICP-cavNS)	Day 88	MTs, H&E, Hart’s stain, IHC (TGF-β1, α-SMA), penile curvature
Castiglione et al, 2019a^22^	SD rats; 8 rats per group; weight 300-350 g; age 12 wk	YES (citrate buffer injection into TA)	Injection of TGF-β1 (0.5 μg/50 μL) into TA, once	Injection of autologous stromal vascular fraction (1 × 10^6^ cells/200 μL) into TA, once on day 1	Day 1	YES (ICP-cavNS)	Day 28	MTs, H&E, WB (collagens, elastin)
Castiglione et al, 2019b^21^	SD rats; 9 rats per group; weight 300-350 g; age 12 wk	YES (citrate buffer injection into TA)	Injection of TGF-β1 (0.5 μg/50 μL) into TA, once	Injection of human adipocyte-derived stem cells (1 × 10^6^ cells) into TA, once on day 28	Day 28	YES (ICP-cavNS)	Day 56	MTs, H&E, WB (collagens, elastin), PCR
Geng et al, 2019^20^	SD rats; 6 rats per group; weight 400-450 g; age not documented	YES (saline injection into TA)	Injection of TGF-β1 (0.5 μg/50 μL) into TA, once	Xiaojin Pill (a Chinese herbal medicine) 107 mg/kg by gavage twice a day for 28 d	Day 0	NO	Day 42	H&E WB (MMP)
Hakim et al, 2019^19^	SD rats; 6 rats per group; weight 300-350 g, age 12 wk	YES (citrate buffer injection into TA)	Injection of TGF-β1 (0.5 μg/50 μL) into TA, once	Injection of autologous stromal vascular fraction (1 × 10^6^ cells/200 μL) into TA, once on day 28	Day 28	YES (ICP-cavNS)	Day 56	MTs, H&E, WB (collagen, elastin)
Ilg et al, 2019^39^	SD rats; 10 rats per group; weight not documented; age 10-12 wk	YES (citrate buffer injection into TA)	Injection of TGF-β1 (1 μg/50 μL) into TA, once	Tamoxifen (5 mg/kg/d, i.p.) with or without vardenafil (1.5 mg/kg/d oral) daily for 5 wk	Day 1	YES (ICP-cavNS)	5 wk	MTs, H&E, WB (α-SMA, collagen) PCR (α-SMA, collagen, elastin)
Antoniassi et al, 2020^18^	Wistar rats; 5 rats per group; weight 469 g; age 90 d	YES (no treatment)	Injection of TGF-β1 (0.5 μg) into TA, on days 0, 3 and 6	Mycophenolate mofetil (immunosuppressant) 30 mg/kg oral once daily for 5 d, 7 and 30 d after TGF-β1 injection	Days 7 and 30	NO	Days 8 and 33	Gomori’s reticulin stain, H&E, penile curvature
Song et al, 2020^17^	SD rats; 6 rats per group; weight not documented; age 4 mo	YES (no treatment)	Injection of mixture of fibrin and thrombin into TA on days 0 and 5.	Vactosertib (ALK5 inhibitor) 10 mg/kg, oral, 5 times a week for 2 wk	Day 0	YES (ICP-cavNS)	Day 30	MTs, H&E, IHC (vimentin, p-Smad2)
Yang et al, 2020^16^	SD rats; 8 rats per group; weight and age not documented	YES (injection of phosphate buffered saline into TA)	Injection of TGF-β1 (0.5 μg in 50 μL) into TA, once	Exosomes obtained from human urine-derived stem cells, 100 μg, injected into TA on day 0	Day 0	YES (ICP-cavNS)	Day 30	MTs, WB (elastin, collagen, α-SMA, p-Smad2/3), PCR (MMPs, TIMPs), ZyA (MMPs)
Cohen et al, 2022^15^	Wistar rats; 5-16 rats per group; weight 300-500 g; age 3 mo	YES (injection of distilled water into TA)	Injection of autologous plasma (20 μL) into TA once or 4 times in 4 consecutive wk	None	N/A	NO	Day 45	Penile curvature, MTs, Picrosirius stain, IHC (heparanase, MMPs, TGF-β1), PCR (heparanase, MMPs, TGF-β1), GAG assay
Wang et al, 2022^14^	SD rats; 8 rats per group; weight 300-350 g; age 11-12 wk	YES (injection of phosphate buffered saline into TA)	Injection of TGF-β1 (50 μg in 50 μL) into TA, on day 0, 1 and 27	Injection of rat bone marrow–derived stem cells (1 × 10^6^ cells in 50 μL) into TA on day 0, 1 and 27	Days 0, 1, and 27	YES (ICP-cavNS)	Day 54	H&E, MTs, IHC (collagen, elastase, Smad7, osteopontin), WB (collagen, elastase, Smad7, osteopontin)
Gundogdu et al., 2023^13^	NZW rabbits; 3 rabbits per group; weight 3.5-4 kg; age not documented	YES (injection of 4 mM hydrochloric acid and 1 mg/mL bovine serum albumin into TA and nonsurgical controls)	Injection of TGF-β1 (0.5 μg in 50 μL) into TA, once	None	N/A	YES (ICP in response to papaverine injection)	1 mo	MTs Picrosirius stain, VVG stain, IHC (collagen, MMPs, TIMPs, elastase)

Abbreviations: α-SMA, α-smooth muscle actin; CC, corpora cavernosa; CD, Clr:CD(SD) rat strain; EM, electron microscopy; GAG, glycosaminoglycan;

H&E, hematoxylin and eosin staining; HP, hydroxyproline;

i.v., intravenous; ICP, intracavernous pressure

ICP-cavNS, intracavernous pressure in response to cavernous nerve electrostimulation;

IFN, interferon;

IHC, immunohistochemistry;

iNOS, inducible nitric oxide synthase;

ivu, infectious virus units;

L-NIL, L-iminoethyl-L-lysine;

MMP, matrix metalloproteinase;

MTs, Masson’s trichrome staining;

N/A, not applicable;

NO, nitric oxide;

NOS, nitric oxide synthase;

NS, nerve stimulation;

NZW, New Zealand White;

p-Smad, phosphorylated Smad;

PCR, polymerase chain reaction;

SD, Sprague Dawley;

Smad2/3, mothers against decapentaplegic homolog 2/3;

TA, tunica albuginea;

TGF-β, transforming growth factor β;

TIMP, tissue inhibitors of metalloproteinase;

VVG, Verhoeff-Van Gieson;

WB, Western blot;

ZyA, Zymography assay.

A total of 40 (90.9%) of 44 studies used a negative control in which the vehicle of the substance was injected into the TA or a sham operation was performed (Table 1). A total of 9 (20.5%) studies repeated the fibrosis inducing agents more than once at various time points. A total of 28 (63.6%) studies utilized the PD animal model to test the preventive (n = 21 [47.7%]) and/or therapeutic (n = 16 [36.4%]) effect of an agent (Table 1). A total of 3 (7%) studies reported a power calculation method by which the number of animals was determined.

A total of 20 (45.5%) studies assessed erectile function either by measuring ICP in response to electrostimulation of the cavernous nerve (n = 19) or to papaverine injection (n = 1). A total of 5 (11.4%) studies measured penile curvature. Out of the measurement methods for fibrosis, the most common was Masson’s trichrome staining (86.4%). The others were immunohistochemistry (47.7%), hematoxylin and eosin staining (45.5%), Hart’s staining (29.5%), Western blotting (27.3%), polymerase chain reaction (PCR) (22.7%), Picrosirius staining (15.9%), electron microscopy (11.4%), Verhoeff-Van Gieson staining (9.1%), zymography assay (6.8%), hydroxyproline assay (6.8%), Safranin-O staining (4.6%), Von Kossa staining (2.3%), glycosaminoglycan assay (2.3%), Gomori staining (2.3%), and magnetic resonance imaging (2.3%) (Table 1).

## Statements

### Statement #1: We suggest adherence to ARRIVE guidelines

#### Evidence and remarks

The ARRIVE (Animal Research: Reporting of In Vivo Experiments) guidelines are a checklist of information to include in publications describing animal research. Any manuscript that describes animal research is expected to submit a completed ARRIVE checklist, which is now mandated by a majority of publishers. Reporting animal research in adherence with the ARRIVE guidelines ensures transparent and thorough reporting that enables the readers and reviewers to scrutinize the research adequately, evaluate its methodological rigor, and reproduce the methods or findings.^65^ A recent study demonstrated that a mandatory ARRIVE checklist during submission is not enough to improve compliance, and more stringent editorial policies are required.^66^ Therefore, adherence to the ARRIVE checklist completion does not necessarily mean that the authors complied with each of items in the guidelines. It should also be noted that the ARRIVE guidelines were introduced in 2010. Out of the 44 studies included in this review, 27 were published after 2010. We were not able to verify whether each of the 27 studies submitted a completed ARRIVE checklist. Although the assessment of these studies for their compliance with the ARRIVE guidelines was not within the scope of this review, the ARRIVE guidelines were utilized as steering points in some of the recommendations made subsequently.

### Statement #2: We suggest reporting the age and weight of the animals at study entry. Weight of the animals at the endpoint should be also reported

#### Evidence

In accordance with the ARRIVE guidelines,^65^ the species, strain, age, and weight of the animals should be reported. All of the 44 studies identified in this review have documented the species and the strain. The age and weight were documented in 65.9% and 45.5% of the 44 studies, respectively. Particularly, it was noted that the age of the rats varied greatly from 2 to 11 months (Table 1).

#### Remarks

The age is particularly pertinent to animal studies in sexual medicine field, as the age is an important determinant of sexual maturity^67^ and aging is known to affect sexual behavior and function.^68^ We suggest using 10- to 12-week-old Sprague Dawley/CD rats, which are known to reach sexual maturity at around that age.^69^ For other species or strains, a literature search should be carried out to find the relevant age when the animal reaches puberty and becomes a sexually mature adult. This information should be included in the paper.

It should also be noted that although PD can occur in any age, the prevalence of the condition has been shown to increase with age,^70^ hence it is more common in men in their 50s and 60s. If PD is to be studied in older age population, rats that are 18 to 24 months old should be utilized.^67^

None of the studies reported the weight of animals at the end of the study. This is particularly important in models in which surgery is involved; any weight loss due to surgery-induced stress should be documented.

### Statement #3: We suggest reporting sample size (total and per group) and sample size calculations

#### Evidence

The ARRIVE guidelines state that the exact number of animals allocated to each group and the total number of animals in each experiment should be specified. How the sample size was decided should be explained; the details of any sample size calculation should be reported.^65^ All 44 studies documented the total number of animals and the number of animals per group. The average number of animals per group was 6.7 ± 2.6 with a wide range of between 3 and 18 (Table 1). Only 3 (6.8%) studies documented sample size calculations.^19^^,^^21^^,^^22^

#### Remarks

Sample size is crucial to assess the validity of the statistical analysis method and the robustness of the results. Sample size calculations should be performed prior to design of the study using freely available software or algorithms. Consultation with a statistician is also suggested. When reporting sample size calculations, the analysis method, the effect size, the estimate of variability, and the power selected should be documented. It should be remembered that low-powered studies are likely to have more false positives and negatives and would risk wasting animals used in inconclusive research.^71^

### Statement #4: We suggest using a negative control group

#### Evidence

According to the ARRIVE guidelines, a control group should be available. If no control group has been used, the rationale should be stated.^65^ Four (9%) of 44 studies did not use a negative control, such as injection of the vehicle substance into TA, and no rationale for the lack of a control group was offered (Table 1).

#### Remarks

A negative control group would ideally be the vehicle of the substance in which the fibrosis inducer (eg, TGF-β1) is dissolved, such as saline or citrate buffer. If the fibrosis is induced by trauma, allograft, shockwave device, or similar nonpharmacological means, a sham control group should be included. The lack of a negative control group without any rationale is concerning because negative control groups are used to determine whether a difference observed in the intervention group is caused by the intervention, not by chance. Without an appropriate negative control group, it cannot be ascertained whether the fibrosis observed in the study is indeed caused by the inducer.

### Statement #5: We suggest providing a detailed description of how PD was induced

#### Evidence

In accordance with the ARRIVE guidelines, for each experimental group, including controls, the authors should describe the procedures in enough detail to allow others to replicate them, including what was done, how it was done, what was used, when and how often, where, and why. Particularly with pharmacological procedures, the details of the following should be given: drug formulation, dose, volume, concentration, site, route and frequency of administration, vehicle or carrier solution formulation and volume, and any evidence that the pharmacological agent used reaches the target tissue. With the surgical procedures, the surgical procedure description, anesthetic used, pre- and postsurgery analgesia, presurgery procedures (eg, fasting), aseptic techniques, monitoring, whether the procedure is terminal or not, duration of the procedure and the anesthesia, and physical variables measured should be reported.^65^

A total of 31 (70.5%) of 44 studies used one of several approaches to elevate TGF-β1 levels in the TA (Table 1). Based on findings that the gene and protein expression of TGF-β1 were increased in the human PD plaque,^72^ Lue and colleagues developed a rat model using an injection of cytomodulin, a TGF-β1–like peptide, into the TA. Six weeks following the injection, the TA of the rats showed a chronic cellular infiltration, elastosis, thickening of the TA, disorganization and clumping of collagen bundles, and expression of TGF-β1.^50^^,^^52^ Later, Bivalacqua et al^48^ reported that injection of the recombinant TGF-β1 protein produced similar effects but that a combined intervention of surgical trauma and TGF-β1 injection caused more profound PD-like changes. Other studies that used TGF-β1–elevating approach utilized adenovirus expressing TGF-β1.^36^^,^^40^

Fibrin was the second most used inducer of fibrosis among the 44 studies that we identified. Five (11.4%) studies used fibrin alone or in combination with TGF-β1 or thrombin to induce fibrosis in TA.^17^^,^^30^^,^^42^^,^^44^^,^^54^ Davila et al^44^ was the first to study the use of fibrin injections into the TA to induce fibrotic plaques in rats. They suggested that in comparison with the TGF-β1 model, the fibrin model better resembled the pathophysiological event of a penile trauma (extravasation of fibrin), which is considered to be the etiological key event for human PD. In addition, the fibrin injection induced a faster plaque formation (2 weeks) and produced larger plaques than the TGF-β1 model. The authors found that the fibrin model reproduced histological features that correlate to human PD: collagen disorganization, fibrin deposition, elastin fragmentation, increased amounts of TGF-β1, inducible nitric oxide synthase, and reactive oxygen species.^42^^,^^44^

Other fibrosis inducers were thrombin only^33^ or in combination with fibrin,^17^^,^^30^^,^^54^ allograft of TA tissue from another rat,^55^ autologous blood^26^ or plasma,^15^ chlorhexidine,^23^ sodium tetradecyl sulfate,^27^^,^^32^ and extracorporeal shockwave (Table 1).^37^

The only animal model that developed PD-like pathology spontaneously was Tsk mice.^38^ The Tsk mouse model is characterized by dermal fibrosis and systemic sclerosis and has recently been shown to develop PD-like lesions.^38^ These mice have a mutation in the gene encoding fibrillin-1, a large ECM structural protein and the major component of microfibrils and display hypodermal fibrosis and some other typical abnormalities of human systemic sclerosis.^73^ The spontaneously occurring PD-like penile changes in Tsk mice include disorganization of the TA structure with accumulation of type I collagen, fibrous plaque formation, penile bending, and areas of chondroid metaplasia with heterotopic ossification.^38^

#### Remarks

The analysis of the data has shown that most of the studies with TGF-β1 injection used 0.5 or 1 μg of the cytokine. There was 1 outlier study that used 50 μg.^14^ We would suggest 0.5 to 1 μg in a small volume such as 50 to 100 μL using a small-gauge needle and microliter syringe. The vehicle in which TGF-β1 is dissolved should be used for the control group.

Although fibrin is the second most common inducer after TGF-β1, its poor solubility and high viscosity can be a technical challenge considering that the volume administered should be no more than 100 μL.

With tissue grafting and blood or plasma, the main issue is standardization of the inducing agents because tissue, blood, and plasma will have inherent variation in their cellular and protein content. This would be amplified in a small volume or space such as rodent TA.

With any of the new techniques, we suggest that the new model should be optimized and standardized in-house and should be reported accordingly. It should always be remembered that any handling, dissection, or surgery to the skin or to the TA is likely to trigger inflammatory response; therefore, appropriate sham control groups should be designed into the study.

Although we do welcome a spontaneous PD animal model, such as the Tsk mouse model, we also acknowledge that this model requires further characterization and optimization. Most importantly the erectile function in Tsk mice should be measured at different ages of the animal so that a correlation between fibrosis development and erectile dysfunction can be assessed.

### Statement #6: We suggest that a detailed description of how fibrosis was measured should be given

#### Evidence

Among the 44 studies that are included in this review, the most common method to measure fibrosis was Masson’s trichrome staining, which was used by 38 (86.4%) studies. The others were immunohistochemistry (47.7%), hematoxylin and eosin staining (45.5%), Hart’s staining (29.5%), Western blotting (27.3%), PCR (22.7%), Picrosirius staining (15.9%), electron microscopy (11.4%), Verhoeff-Van Gieson staining (9.1%), zymography assay (6.8%), hydroxyproline assay (6.8%), Safranin-O staining (4.6%), Von Kossa staining (2.3%), glycosaminoglycan assay (2.3%), Gomori staining (2.3%), and magnetic resonance imaging (2.3%) (Table 1). The reporting of the details of methods varied among the techniques used as well as among the studies. It should be noted that we have not included in our assessment those methods that measure project-specific endpoints (ie, nitric oxide synthase activity) rather than fibrosis.

#### Remarks

The methods that were employed in measurement of fibrosis in the 44 publications that we have reviewed are listed in Table 2 in which we summarized the principles of the methods as well as their advantages and disadvantages.

**Table 2:** Summary of fibrosis measurement methods used in Peyronie’s disease animal models.

**Method**	**What does it measure?**	**Advantages**	**Disadvantages**
Masson’s trichrome staining	Nuclei and other basophilic structures are stained blue. Cytoplasm, muscle, erythrocytes and keratin are stained red. Collagen is stained green or blue, depending on which variant of the technique is used. In TA tissue, it stains smooth muscle (red) and fibrotic tissue (blue).	Inexpensive Easy Can be used to measure overall extent of fibrosis	Semi-quantitative Requires blinding during quantification
Immunohistochemistry	Visualization of protein expression in tissue sections.	Relatively easy Commonly used in research labs Can be used to assess the changes in expression of proteins	Expensive due to antibody costs Semi-quantitative Quantifiable with appropriate internal control Antibodies should be validated in-house
Hematoxylin and eosin staining	Nuclei are stained blue, cytoplasm and extracellular matrix are stained pink.	Inexpensive Easy Commonly used in pathology labs Can be used to assess the overall structural changes	Does not measure fibrosis Semi-quantitative Requires blinding during quantification
Hart’s staining	Elastic fibers are stained black-dark blue, nuclei are stained dark black-blue, collagen is stained red-pink, other tissue elements are stained yellow.	Inexpensive Easy Can be used to assess the overall changes to elastic fibers	Only elastic fibers Semi-quantitative Requires blinding during quantification
Western blotting	Measures expression of proteins.	Commonly used in research labs Relatively easy Can be used to assess the overall changes in protein expression	Expensive due to antibody costs Semi-quantitative Equal amount of protein should be loaded Quantifiable with appropriate internal control Antibodies should be validated in-house
PCR	Measures expression of mRNA.	Commonly used technique in research labs Relatively easy Can be used to assess the overall changes in protein expression Can be quantitative	Expensive due to consumables costs RNA quality and PCR methods including reverse transcription should be validated in-house International MIQE guidelines should be followed
Picrosirius red staining	Stains collagen I and III fibers.	Relatively easy Can be used to assess the overall changes in collagen I and III fibers	Only 2 types of collagen fibers Semi-quantitative Requires blinding during quantification Uses picric acid, which can pose explosion hazard when it dries
Electron microscopy	Visualizes cellular structures at nanometer scale.	Can be used to assess the changes in tissue and cell architecture	Expensive Not widely available Nonquantitative
Verhoeff-Van Gieson staining	Elastic fibers and nuclei are stained black, collagen fibers are stained red, and cytoplasmic elements are stained yellow.	Inexpensive Easy Can be used to assess the overall changes to elastic fibers	Mostly to visualize elastic fibers Semi-quantitative Requires blinding during quantification Uses picric acid which can pose explosion hazard when it dries
Zymography assay	Measures activity of hydrolases and proteinases.	Not a common technique Can be used to measure overall changes in metalloproteinease activity	Would require equal amount of sample loading Needs in-house assay optimization Semi-quantitative
Hydroxyproline assay	Measures amount of hydroxyproline.	Inexpensive Easy Quantitative Can be used to measure overall changes in hydroxyproline amount as an indirect measure of collagen amount	Would not distinguish between collagen types Would require equal amount of sample loading Needs in-house assay optimization
Safranin-O staining	Nuclei are stained black, cartilage, mucin and mast cells are stained orange to red.	Inexpensive Easy Can be used to assess cartilage formation	Only cartilage Semi-quantitative Requires blinding during quantification
Von Kossa staining	Nuclei are stained red, calcium salts are stained black or brown-black, cytoplasm is stained pink.	Inexpensive Easy Can be used to assess calcium deposition	Only calcium deposits Semi-quantitative Requires blinding during quantification
GAG assay	Measures amount of GAGs.	Inexpensive Easy Quantitative Can be used to measure overall changes in GAGs as an indirect measure of ECM content	Would not distinguish between ECM types Would require equal amount of sample loading Needs in-house assay optimization
Gomori staining	Stains muscle fibers and cytoplasm in red, nuclei in black, and collagen-containing connective tissues in blue.	Inexpensive Easy Can be used to stain reticular and collagen fibers	Semi-quantitative Requires blinding during quantification
Magnetic resonance imaging	Using magnetic fields and radio waves produces detailed images of the body and organs.	Can be used to assess the changes in organ architecture	Expensive Not widely available Not sufficient resolution at tissue or cell level

Abbreviations: ECM, extracellular matrix; GAG, glycosaminoglycan; MIQE, Minimum Information for Publication of Quantitative Real-Time PCR Experiments; mRNA, messenger RNA; PCR, polymerase chain reaction; TA, tunica albuginea.

Based on the previous assessment, we would suggest that Masson’s trichome method should be utilized to measure the smooth muscle/fibrosis ratio. Any quantification from Masson’s trichrome staining should be performed blindly (ie, by a person who is not aware which experimental group the samples belong to) in multiple regions of interest from multiple slides for each experimental group, ideally using an image analysis software. The researchers should also note that because this staining method is not protein specific, erythrocytes would for example be stained red like smooth muscle cells.

Our second suggestion would be to quantify the protein amount of ECMs and smooth muscle. Ideally this should be performed using Western blotting or enzyme-linked immunosorbent assay to measure relevant collagens (eg, collagen I and III), elastin, and α-smooth muscle actin. The source, catalog number and titers of the antibodies should be reported. The results should be normalized to an internal protein that is known not to be altered by fibrosis such as GAPDH.^74^ Ratios between collagens/elastin and smooth muscle should be calculated and reported.

We suggest Masson’s trichrome staining and protein quantification as the minimum requirement for fibrosis evaluation. The other methods listed in Table 2 can also be employed depending on the research question and the project-specific endpoints. Quantitative PCR methods such as real-time quantitative PCR can be employed, as long as international MIQE (Minimum Information for Publication of Quantitative Real-Time PCR Experiments) guidelines^75^ are followed, to obtain quantitative data with low variability. Any results from PCR should be validated using protein expression since not all changes in messenger RNA expression necessarily translate to changes in protein expression.^76^^,^^77^

### Statement #7: A PD model does not need to cause penile curvature or calcification in rats

#### Evidence

Of 44 publications we have identified, 5 measured penile curvature in rats.^15^^,^^18^^,^^23^^,^^40^^,^^55^ Curvature was measured typically by measuring the angle of curvature in the photos with a protractor. Artificial erection using saline or water injection into the cavernous space was often employed before the angle was measured. Three studies assessed cartilage formation or calcification^36^^,^^40^^,^^55^ using Safranin-O or Von Kossa staining.

#### Remarks

Rat penis shows distinct differences in anatomy and physiology compared with human penis. It has a cylindrical shaft and a bulbous glans with 90° frontal flex. It has also a cylindrical bone (*os penis*) that extends from the distal end of the body to the tip of the glans.^78^ Striated muscle contraction is required to straighten the glans during intromission.^79^ Despite this uniquely different anatomy and physiology, rats are frequently used in research involving erectile function/dysfunction and PD due to the presence of human-like corpus cavernosum, TA, low cost, and relatively larger size than mice. For example, ICP measurements in rats in response to cavernous nerve stimulation have now been widely accepted standard model for erectile function assessment.^63^

When a fibrosis inducing agent such as TGF-β1 or fibrin is injected to the shaft of the rat penis, a fibrotic plaque does indeed form. But it would be practically challenging to accurately measure the angle of any curvature formed by this plaque simply because of the anatomy of the rat penis. We suggest that such curvature measurements can be used to follow up the development of the plaque but should not be used as a primary end point. Similarly, fibrosis in rat TA does not always produce calcification and/or cartilage/bone formation. When it does, the amounts are too small to measure and quantify accurately. We suggest that the measures of calcification, cartilage/bone formation should not be used as primary end points.

### Statement #8: We suggest measuring erectile function using cavernous nerve electrostimulation in PD animal models

#### Evidence

A total of 20 (45.5%) studies assessed erectile function either by measuring ICP in response to electrostimulation of the cavernous nerve (n = 19) or to papaverine injection (n = 1) (Table 1). Particularly quantitative ICP: mean arterial pressure (MAP) ratios were successfully utilized to measure the effect and test hypotheses.

#### Remarks

Although not all patients with PD develop ED, erectile function measurement has proven to be a useful functional and quantitative proxy for PD in animal models. We suggest that ICP and MAP measurements should be performed under anesthesia; the ICP and MAP values and their ratios should be reported. Ideally representative tracings of ICP and MAP should also be presented. The erections should be induced by electrostimulation, rather than by vasoactive agents like papaverine, as fibrosis may alter the local pharmacokinetics of injected drugs. For other technical details of this procedure, the ESSM guidelines should be followed.^63^

### Statement #9: We suggest considering spontaneous recovery of fibrosis in the experimental design

#### Evidence

Of 44 studies reviewed here, 29 (65.9%) tested a treatment. Of those 29 studies, 14 (48.3%) tested a treatment to prevent fibrosis meaning that the treatment was initiated on the day of or shortly after fibrosis induction (Table 1). Seven (24.1%) studies tested a treatment to reverse fibrosis, meaning that the treatment was initiated once the fibrosis was established (Table 1). Eight (27.6%) studies tested a treatment in both prevention and reversal mode, meaning that the treatment was initiated immediately after the induction of fibrosis in one group of animals and initiated once the fibrosis was established in another group of animals (Table 1). In reversal mode, the treatment was initiated on average ~30 days after the fibrosis induction. The delay between the fibrosis induction and the start of the treatment varied largely between 15 and 45 days (Table 1).

#### Remarks

To test disease preventing agents, initiation of the treatment immediately or 1 to 2 days after the fibrosis induction (eg, TGF-β1 or fibrin) is highly suggested. The importance and necessity of a time-matched control group should be highlighted once again here because it would not be possible to draw any conclusion on the effectiveness of preventive treatment without such a control group.

The researchers should note that experimentally induced fibrosis in animal models may spontaneously resolve without any treatment as shown in bleomycin-induced pulmonary fibrosis,^80^ systemic sclerosis^81^ in mice, and TGF-β1-induced PD in rats.^21^ It is therefore essential to have time-matched control groups. Pilot experiments should be performed to find the optimum delay time and the time for the study end; the results from these pilot studies should be reported.

A temporal analysis of Tsk model should be conducted to investigate whether fibrosis in TA of these mice undergo spontaneous resolution.

From the clinical translation point of view, the preventive treatment groups will be investigating the effect of treatment modalities targeting early-stage (acute phase) PD. The investigation into treatment modalities that target late-stage (chronic phase) PD is made difficult by the spontaneous resolution of fibrosis in animal models.

## Conclusion

Several animal models for PD are available, but unfortunately none of the models mimic human PD precisely. The animal models reviewed here are based on different approaches to induce PD-like changes, and each model may to some extent simulate 1 or more mechanisms or signs of the human pathology. To increase the validity and translatability of PD research in animal models, it is crucial that each of the models is methodologically standardized and optimized; doing so ensures that the models mimic the human physiology as closely as possible. In this document, we provided ESSM position statements for the correct and reproducible use of these animal models in PD research. We hope that the suggestions we made here will advance the PD field and will narrow the translational gap.

## Funding

None declared.

*Conflict of interest:* None declared.

## References

[1] Hellstrom WJG . History, epidemiology, and clinical presentation of Peyronie’s disease. Int J Impot Res. 2003;15(Suppl 5):S91–S92. 10.1038/sj.ijir.3901081. 14551584

[2] Mulhall JP , Creech SD, Boorjian SA, et al. Subjective and objective analysis of the prevalence of Peyronie’s disease in a population of men presenting for prostate cancer screening. J Urol. 2004;171(6 Pt 1):2350–2353. 10.1097/01.ju.0000127744.18878.f1. 15126819

[3] Lindsay MB , Schain DM, Grambsch P, Benson RC, Beard CM, Kurland LT. The incidence of Peyronie’s disease in Rochester, Minnesota, 1950 through 1984. J Urol. 1991;146(4):1007–1009. 10.1016/S0022-5347(17)37988-0. 1895413

[4] Mulhall JP , Schiff J, Guhring P. An analysis of the natural history of Peyronie’s disease. J Urol. 2006;175(6):2115–2118. 10.1016/S0022-5347(06)00270-9. 16697815

[5] Hatzimouratidis K , Eardley I, Giuliano F, et al. EAU guidelines on penile curvature. Eur Urol. 2012;62(3):543–552. 10.1016/j.eururo.2012.05.040. 22658761

[6] Nelson CJ , Diblasio C, Kendirci M, Hellstrom W, Guhring P, Mulhall JP. The chronology of depression and distress in men with Peyronie’s disease. J Sex Med. 2008;5(8):1985–1990. 10.1111/j.1743-6109.2008.00895.x. 18554257

[7] Moreland RB , Nehra A. Pathophysiology of Peyronie’s disease. Int J Impot Res. 2002;14(5):406–410. 10.1038/sj.ijir.3900875. 12454693

[8] Gonzalez-Cadavid NF , Rajfer J. Mechanisms of disease: new insights into the cellular and molecular pathology of Peyronie’s disease. Nat Clin Pract Urol. 2005;2(6):291–297. 10.1038/ncpuro0201. 16474811

[9] Hauck EW , Hauptmann A, Schmelz HU, Bein G, Weidner W, Hackstein H. Prospective analysis of single nucleotide polymorphisms of the transforming growth factor beta-1 gene in Peyronie’s disease. J Urol. 2003;169(1):369–372. 10.1097/01.ju.0000039347.38539.13. 12478192

[10] Nugteren HM , Nijman JM, de Jong IJ, van Driel MF. The association between Peyronie’s and Dupuytren’s disease. Int J Impot Res. 2011;23(4):142–145. 10.1038/ijir.2011.18. 21633367

[11] Gelfand RA , Vernet D, Kovanecz I, Rajfer J, Gonzalez-Cadavid NF. The transcriptional signatures of cells from the human Peyronie’s disease plaque and the ability of these cells to generate a plaque in a rat model suggest potential therapeutic targets. J Sex Med. 2015;12(2):313–327. 10.1111/jsm.12760. 25496134 PMC4339076

[12] Jalkut M , Gonzalez-Cadavid N, Rajfer J. New discoveries in the basic science understanding of Peyronie’s disease. Curr Urol Rep. 2004;5(6):478–484. 10.1007/s11934-004-0074-y. 15541219

[13] Gundogdu G , Nguyen T, Namasivayam A, Starek S, Gelman J, Mauney JR. Characterization of a novel rabbit model of Peyronie’s disease. Int J Impot Res. 2023; 10.1038/s41443-023-00671-y. PMC11035118 36782023

[14] Wang W , Ding W, Zhang X, et al. Intratunical injection of rat-derived bone marrow mesenchymal stem cells prevents fibrosis and is associated with increased Smad7 expression in a rat model of Peyronie’s disease. Stem Cell Res Ther. 2022;13(1):390. 10.1186/s13287-022-03090-w. 35908015 PMC9338499

[15] Cohen DJ , Reynaldo WV, Borba VB, et al. New in vivo model to assess macroscopic, histological, and molecular changes in Peyronie’s disease. Andrology. 2022;10(1):154–165. 10.1111/andr.13092. 34464514

[16] Yang Q , Chen W, Han D, et al. Intratunical injection of human urine-derived stem cells derived exosomes prevents fibrosis and improves erectile function in a rat model of Peyronie’s disease. Andrologia. 2020;52(11):e13831. 10.1111/and.13831. 32986908

[17] Song KM , Chung DY, Choi MJ, et al. Vactosertib, a novel, orally bioavailable activin receptor-like kinase 5 inhibitor, promotes regression of fibrotic plaques in a rat model of Peyronie’s disease. World J Mens Health. 2020;38(4):552–563. 10.5534/wjmh.190071. 31496148 PMC7502315

[18] Antoniassi T , Júnior FNF, Spessoto LCF, Guerra LH, Campos SS, Taboga S. Anti-fibrotic effect of mycophenolate mofetil on Peyronie’s disease experimentally induced with TGF-β. Int J Impot Res. 2020;32(2):201–206. 10.1038/s41443-019-0138-7. 31000815

[19] Hakim L , Fiorenzo S, Hedlund P, et al. Intratunical injection of autologous adipose stromal vascular fraction reduces collagen III expression in a rat model of chronic penile fibrosis. Int J Impot Res. 2020;32(3):281–288. 10.1038/s41443-019-0136-9. 30988428

[20] Geng Q , Wang F, Han Q, et al. Antioxidant mechanism of Xiaojin pill () for treatment of Peyronie’s disease in rats based on matrix metalloproteinases. Chin J Integr Med. 2019;25(9):671–676. 10.1007/s11655-019-3203-7. 31650486

[21] Castiglione F , Hedlund P, Weyne E, et al. Intratunical injection of human adipose tissue-derived stem cells restores collagen III/I ratio in a rat model of chronic Peyronie’s disease. Sex Med. 2019;7(1):94–103. 10.1016/j.esxm.2018.09.003. 30503767 PMC6377372

[22] Castiglione F , Hedlund P, Weyne E, et al. Intratunical injection of stromal vascular fraction prevents fibrosis in a rat model of Peyronie’s disease. BJU Int. 2019;124(2):342–348. 10.1111/bju.14570. 30267556

[23] Jiang H , Gao Q, Che X, et al. Repeated micro-trauma of the penile tunica albuginea: a new animal model of Peyronie’s disease. Urol Int. 2018;100(2):228–239. 10.1159/000475601. 29151107

[24] Culha MG , Erkan E, Cay T, Yücetaş U. The effect of platelet-rich plasma on Peyronie’s disease in rat model. Urol Int. 2019;102(2):218–223. 10.1159/000492755. 30317233

[25] Li J , Wang S, Qin F, et al. Reduction in Peyronie’s-like plaque size using a vacuum erection device in a rat model of Peyronie’s disease via the TGF-β/SMAD signalling pathway. Andrologia. 2018;50(7):e13051. 10.1111/and.13051. 29806152

[26] Cohen DJ , Oliveira AV, Theodoro TR, et al. Extracellular matrix alterations after blood instillation in tunica albuginea of rats. Int J Impot Res. 2018;30(2):85–92. 10.1038/s41443-017-0015-1. 29242634

[27] Lin H , Liu C, Wang R. Effect of penile traction and vacuum erectile device for Peyronie’s disease in an animal model. J Sex Med. 2017;14(10):1270–1276. 10.1016/j.jsxm.2017.08.011. 28923308

[28] Kaya E , Kibar Y, Yilmaz S, et al. The histopathological effects of intracavernosal mitomycin-C injection in a rat Peyronie’s disease model. Can Urol Assoc J. 2017;11(11):E441–E445. 10.5489/cuaj.4489. 29072564 PMC5698024

[29] Gokce A , Abd Elmageed ZY, Lasker GF, et al. Intratunical injection of genetically modified adipose tissue-derived stem cells with human interferon α-2b for treatment of erectile dysfunction in a rat model of tunica Albugineal fibrosis. J Sex Med. 2015;12(7):1533–1544. 10.1111/jsm.12916. 26062100 PMC4914049

[30] Sohn DW , Bae WJ, Kim HS, Kim SW, Kim SW. The anti-inflammatory and antifibrosis effects of anthocyanin extracted from black soybean on a Peyronie disease rat model. Urology. 2014;84(5):1112–1116. 10.1016/j.urology.2014.06.026. 25156514

[31] Gokce A , Abd Elmageed ZY, Lasker GF, et al. Adipose tissue-derived stem cell therapy for prevention and treatment of erectile dysfunction in a rat model of Peyronie’s disease. Andrology. 2014;2(2):244–251. 10.1111/j.2047-2927.2013.00181.x. 24574095

[32] Chung E , Garcia F, de Young L, Solomon M, Brock GB. A comparative study of the efficacy of intralesional verapamil versus normal saline injection in a novel Peyronie disease animal model: assessment of immunohistopathological changes and erectile function outcome. J Urol. 2013;189(1):380–384. 10.1016/j.juro.2012.08.191. 23174244

[33] Cerruto MA , D’Elia C, Molinari A, Cavicchioli FM, D’Amico A, Artibani W. Animal experimental model of Peyronie’s disease: a pilot study. Arch Ital Urol Androl. 2013;85(1):28–33. 10.4081/aiua.2013.1.28. 23695402

[34] Castiglione F , Hedlund P, van der Aa F, et al. Intratunical injection of human adipose tissue-derived stem cells prevents fibrosis and is associated with improved erectile function in a rat model of Peyronie’s disease. Eur Urol. 2013;63(3):551–560. 10.1016/j.eururo.2012.09.034. 23040209 PMC4029115

[35] Akman T , Tefekli A, Armagan A, et al. Decorin as a new treatment alternative in Peyronie’s disease: preliminary results in the rat model. Andrologia. 2013;45(2):101–106. 10.1111/j.1439-0272.2012.01318.x. 22670875

[36] Ryu J-K , Piao S, Shin H-Y, et al. IN-1130, a novel transforming growth factor-beta type I receptor kinase (activin receptor-like kinase 5) inhibitor, promotes regression of fibrotic plaque and corrects penile curvature in a rat model of Peyronie’s disease. J Sex Med. 2009;6(5):1284–1296. 10.1111/j.1743-6109.2009.01216.x. 19473283

[37] Andrade E , Cortez I, Claro J, et al. Preliminary findings from a new animal model for Peyronie’s disease involving extracorporeal shockwaves. BJU Int. 2009;103(8):1104–1106. 10.1111/j.1464-410X.2008.08173.x. 19021616

[38] Lucattelli M , Lunghi B, Fineschi S, et al. A new mouse model of Peyronie’s disease: an increased expression of hypoxia-inducible factor-1 target genes during the development of penile changes. Int J Biochem Cell Biol. 2008;40(11):2638–2648. 10.1016/j.biocel.2008.05.012. 18599338

[39] Ilg MM , Mateus M, Stebbeds WJ, et al. Antifibrotic synergy between phosphodiesterase type 5 inhibitors and selective oestrogen receptor modulators in Peyronie’s disease models. Eur Urol. 2019;75(2):329–340. 10.1016/j.eururo.2018.10.014. 30344087

[40] Piao S , Ryu J-K, Shin H-Y, et al. Repeated intratunical injection of adenovirus expressing transforming growth factor-beta1 in a rat induces penile curvature with tunical fibrotic plaque: a useful model for the study of Peyronie’s disease. Int J Androl. 2008;31(3):346–353. 10.1111/j.1365-2605.2007.00780.x. 17651407

[41] Ferrini MG , Kovanecz I, Nolazco G, Rajfer J, Gonzalez-Cadavid NF. Effects of long-term vardenafil treatment on the development of fibrotic plaques in a rat model of Peyronie’s disease. BJU Int. 2006;97(3):625–633. 10.1111/j.1464-410X.2006.05955.x. 16469038

[42] Davila HH , Magee TR, Vernet D, Rajfer J, Gonzalez-Cadavid NF. Gene transfer of inducible nitric oxide synthase complementary DNA regresses the fibrotic plaque in an animal model of Peyronie’s disease. Biol Reprod. 2004;71(5):1568–1577. 10.1095/biolreprod.104.030833. 15240426

[43] Valente EGA , Vernet D, Ferrini MG, Qian A, Rajfer J, Gonzalez-Cadavid NF. L-arginine and phosphodiesterase (PDE) inhibitors counteract fibrosis in the Peyronie’s fibrotic plaque and related fibroblast cultures. Nitric Oxide. 2003;9(4):229–244. 10.1016/j.niox.2003.12.002. 14996430

[44] Davila HH , Ferrini MG, Rajfer J, Gonzalez-Cadavid NF. Fibrin as an inducer of fibrosis in the tunica albuginea of the rat: a new animal model of Peyronie’s disease. BJU Int. 2003;91(9):830–838. 10.1046/j.1464-410x.2003.04224.x. 12780843

[45] Vernet D , Ferrini MG, Valente EG, et al. Effect of nitric oxide on the differentiation of fibroblasts into myofibroblasts in the Peyronie’s fibrotic plaque and in its rat model. Nitric Oxide. 2002;7(4):262–276. 10.1016/s1089-8603(02)00124-6. 12446175

[46] Ferrini MG , Vernet D, Magee TR, et al. Antifibrotic role of inducible nitric oxide synthase. Nitric Oxide. 2002;6(3):283–294. 10.1006/niox.2001.0421. 12009846

[47] Bivalacqua TJ , Champion HC, Leungwattanakij S, et al. Evaluation of nitric oxide synthase and arginase in the induction of a Peyronie’s-like condition in the rat. J Androl. 2001;22(3):497–506. 11330651

[48] Bivalacqua TJ , Diner EK, Novak TE, et al. A rat model of Peyronie’s disease associated with a decrease in erectile activity and an increase in inducible nitric oxide synthase protein expression. J Urol. 2000;163(6):1992–1998. 10799245

[49] El-Sakka AI , Bakircioglu ME, Bhatnagar RS, Yen TS, Dahiya R, Lue TF. The effects of colchicine on a Peyronie’s-like condition in an animal model. J Urol. 1999;161(6):1980–1983. 10332485

[50] El-Sakka AI , Hassan MU, Nunes L, Bhatnagar RS, Yen TS, Lue TF. Histological and ultrastructural alterations in an animal model of Peyronie’s disease. Br J Urol. 1998;81(3):445–452. 10.1046/j.1464-410x.1998.00529.x. 9523668

[51] El-Sakka AI , Selph CA, Yen TS, Dahiya R, Lue TF. The effect of surgical trauma on rat tunica albuginea. J Urol. 1998;159(5):1700–1707. 10.1097/00005392-199805000-00097. 9554397

[52] El-Sakka AI , Hassoba HM, Chui RM, Bhatnagar RS, Dahiya R, Lue TF. An animal model of Peyronie’s-like condition associated with an increase of transforming growth factor beta mRNA and protein expression. J Urol. 1997;158(6):2284–2290. 10.1016/s0022-5347(01)68236-3. 9366377

[53] Cantini LP , Ferrini MG, Vernet D, et al. Profibrotic role of myostatin in Peyronie’s disease. J Sex Med. 2008;5(7):1607–1622. 10.1111/j.1743-6109.2008.00847.x. 18422491

[54] Kwon K-D , Choi MJ, Park J-M, et al. Silencing histone deacetylase 2 using small hairpin RNA induces regression of fibrotic plaque in a rat model of Peyronie’s disease. BJU Int. 2014;114(6):926–936. 10.1111/bju.12812. 24841412

[55] Ferretti L , Fandel TM, Qiu X, et al. Tunica albuginea allograft: a new model of LaPeyronie’s disease with penile curvature and subtunical ossification. Asian J Androl. 2014;16(4):592–596. 10.4103/1008-682X.125900. 24759578 PMC4104088

[56] Milenkovic U , Ilg M, Cellek S, Albersen M. What role do pharmaceuticals play in the treatment of Peyronie’s disease and is there a need for new emerging drugs? Expert Opin Emerg Drugs. 2019;24(1):1–4. 10.1080/14728214.2019.1591370. 30845848

[57] Milenkovic U , Ilg MM, Cellek S, Albersen M. Pathophysiology and future therapeutic perspectives for resolving fibrosis in Peyronie’s disease. Sex. Med Rev. 2019;7(4):679–689. 10.1016/j.sxmr.2019.02.004. 30962046

[58] García-Gómez B , Aversa A, Alonso-Isa M, et al. The use of penile traction devices for Peyronie’s disease: position statements from the European Society for Sexual Medicine. Sex Med. 2021;9(4):100387. 10.1016/j.esxm.2021.100387. 34273788 PMC8360933

[59] Osmonov D , Ragheb A, Ward S, et al. ESSM position statement on surgical treatment of Peyronie’s disease. Sex Med. 2022;10(1):100459. 10.1016/j.esxm.2021.100459. 34823053 PMC8847818

[60] Russo GI , Milenkovic U, Hellstrom W, Levine LA, Ralph D, Albersen M. Clinical efficacy of injection and mechanical therapy for Peyronie’s disease: a systematic review of the literature. Eur Urol. 2018;74(6):767–781. 10.1016/j.eururo.2018.07.005. 30237020

[61] Megson M , Merrett C, Ilg MM, Cellek S, Ralph D. Can tamoxifen and a PDE5 inhibitor slow the progression of Peyronie’s disease? J Sex Med. 2022;19(11):S15–S16. 10.1016/j.jsxm.2022.08.158.

[62] Geelhoed JP , de Jong IJ, Beck JJH. Treatment of acute phase of Peyronie’s disease with tamoxifen and vardenafil/tadalafil off label. J Sex Med. 2023;20(Suppl 4):qdad062.122.

[63] Weyne E , Ilg MM, Cakir OO, et al. European Society for Sexual Medicine consensus statement on the use of the cavernous nerve injury rodent model to study postradical prostatectomy erectile dysfunction. Sex Med. 2020;8(3):327–337. 10.1016/j.esxm.2020.06.007. 32674971 PMC7471127

[64] Guyatt GH , Oxman AD, Vist GE, et al. GRADE: an emerging consensus on rating quality of evidence and strength of recommendations. BMJ. 2008;336(7650):924–926. 10.1136/bmj.39489.470347.AD. 18436948 PMC2335261

[65] Percie du Sert N , Hurst V, Ahluwalia A, et al. The ARRIVE guidelines 2.0: updated guidelines for reporting animal research. PLoS Biol. 2020;18(7):e3000410. 10.1371/journal.pbio.3000410. 32663219 PMC7360023

[66] Hair K , Macleod MR, Sena ES. A randomised controlled trial of an intervention to improve compliance with the ARRIVE guidelines (IICARus). Res Integr Peer Rev. 2019;4(1):12. 10.1186/s41073-019-0069-3. 31205756 PMC6560728

[67] Sengupta P . The laboratory rat: relating its age with human’s. Int J Prev Med. 2013;4(6):624–630. 23930179 PMC3733029

[68] Smith ER , Stefanick ML, Clark JT, Davidson JM. Hormones and sexual behavior in relationship to aging in male rats. Horm Behav. 1992;26(1):110–135. 10.1016/0018-506x(92)90035-t. 1563724

[69] Fuochi S , Galasso ME, Colombo R, Giaquinto D, de Girolamo P, D’Angelo L. Puberty onset curve in CD (Sprague Dawley) and long Evans outbred male rats. Lab Anim (NY). 2022;56(5):471–475. 10.1177/00236772221078725. 35253499

[70] Ostrowski KA , Gannon JR, Walsh TJ. A review of the epidemiology and treatment of Peyronie’s disease. Res Rep Urol. 2016;8:61–70. 10.2147/RRU.S65620. 27200305 PMC4857830

[71] Würbel H . More than 3Rs: the importance of scientific validity for harm-benefit analysis of animal research. Lab Anim (NY). 2017;46(4):164–166. 10.1038/laban.1220. 28328898

[72] El-Sakka AI , Hassoba HM, Pillarisetty RJ, Dahiya R, Lue TF. Peyronie’s disease is associated with an increase in transforming growth factor-beta protein expression. J Urol. 1997;158(4):1391–1394. 9302128

[73] Christner PJ , Jimenez SA. Animal models of systemic sclerosis: insights into systemic sclerosis pathogenesis and potential therapeutic approaches. Curr Opin Rheumatol. 2004;16(6):746–752. 10.1097/01.bor.0000137893.68929.86. 15577614

[74] Ilg MM , Stafford SJ, Mateus M, et al. Phosphodiesterase type 5 inhibitors and selective estrogen receptor modulators can prevent but not reverse myofibroblast transformation in Peyronie’s disease. J Sex Med. 2020;17(10):1848–1864. 10.1016/j.jsxm.2020.06.022. 32771352

[75] Bustin SA , Benes V, Garson JA, et al. The MIQE guidelines: minimum information for publication of quantitative real-time PCR experiments. Clin Chem. 2009;55(4):611–622. 10.1373/clinchem.2008.112797. 19246619

[76] Schwanhäusser B , Busse D, Li N, et al. Global quantification of mammalian gene expression control. Nature. 2011;473(7347):337–342. 10.1038/nature10098. 21593866

[77] Tian Q , Stepaniants SB, Mao M, et al. Integrated genomic and proteomic analyses of gene expression in mammalian cells. Mol Cell Proteomics. 2004;3(10):960–969. 10.1074/mcp.M400055-MCP200. 15238602

[78] Goyal HO , Braden TD, Williams CS, et al. Abnormal morphology of the penis in male rats exposed neonatally to diethylstilbestrol is associated with altered profile of estrogen receptor-α protein, but not of androgen receptor protein: a developmental and immunocytochemical study. Biol Reprod. 2004;70(5):1504–1517. 10.1095/biolreprod.103.026328. 14749301

[79] Sachs BD . Role of striated penile muscles in penile reflexes, copulation, and induction of pregnancy in the rat. J Reprod Fertil. 1982;66(2):433–443. 10.1530/jrf.0.0660433. 7175800

[80] Tashiro J , Rubio GA, Limper AH, et al. Exploring animal models that resemble idiopathic pulmonary fibrosis. Front Med (Lausanne). 2017;4:118. 10.3389/fmed.2017.00118. 28804709 PMC5532376

[81] Artlett C . Animal models of systemic sclerosis: their utility and limitations. *Open Access*. Rheumatol. 2014;6:65–81. 10.2147/OARRR.S50009. PMC5045111 27790036

